# Use, knowledge, and effectiveness of nutritional traffic light label in an urban population from Ecuador: a pilot study

**DOI:** 10.1186/s12992-019-0467-9

**Published:** 2019-03-29

**Authors:** Santiago Teran, Isabel Hernandez, Wilma Freire, Beatriz Leon, Enrique Teran

**Affiliations:** 1 Colegio de Ciencias de la Salud, Universidad San Francisco de Quito, Quito, Ecuador; 2 Facultad de Enfermeria, Pontificia Universidad Católica del Ecuador, Quito, Ecuador; 3 Instituto de Salud y Nutrición, Universidad San Francisco de Quito, Quito, Ecuador

**Keywords:** Nutritional traffic light label, Nutritional labels, Overweight, Obesity, Non-communicable diseases

## Abstract

**Background:**

The nutritional traffic light label (NTLL) has become one of the most used Front of Package labels (FOP’s) around the world, for its simple and easy to understand graphical system. In Ecuador, this labelling system has recently been implemented; then, this research aims to evaluate the use and knowledge of NTLL and its effectiveness as a public health promotion strategy.

**Method:**

In a cross-sectional study at two different urban supermarkets in Quito-Ecuador, a survey was conducted in 73 participants to inquire about knowledge, perspectives and purchasing habits regarding the NTLL. Objective data obtained from pictures of the participants’purchase was compared with subjective data obtained from the survey. For categorical variables, Chi square or Fisher’s Exact test were used and variables with a statistical significance at α = 0.1 were included in multivariate logistic regression models.

**Results:**

88.7% of participants knew about the NTTL. 27.4% reported using the NTLL, while 28.4% of participants were observed to really use it. Significant associations between self-knowledge of the NTLL and education level (*p* = 0.007) or knowledge level (*p* = 0.001) were found. A significant association was also found between the refered use of the NTLL and the shopping influencing factor (*p* = 0.02). In the multivariate analysis an association between knowledge of the NTLL and observed use was found only when adjusted for the supermarket (*p* = 0.038).

**Conclusion:**

This study found that the level of knowledge of the NTLL in the studied population was relatively high; however, both the referred and the observed use of the NTLL were low. Use and knowledge of the NTLL were associated with the socioeconomic and educational status of the participants. Thus, the change in nutritional patterns needs additional strategies to put the NTLL before the brand once customers make their purchases.

## Introduction

Obesity prevalence worldwide almost doubled between 1980 and 2014, making it a serious public health problem [1]. According to the World Health Organization (WHO), by 2014, 39% of adults 18 years and older were overweight. According to United Nations International Children’s Emergency Fund (UNICEF), the overall prevalence of overweight and obesity in children under 5 years of age has increased from about 5% in 2000 to 6% in 2010 and 6.3% by 2013 [1].

Two key factors have contributed to the increase in the prevalence of obesity: a deficient access to public health information, as well as, an increased exposure to highly processed foods [2]. Unfortunately, overweight and obesity do not act as particular pathologies, however, they correlate and lead to different comorbidities among non-communicable diseases [3].

Regarding Ecuador, the WHO describes a prevalence of overweight and obesity for adults of 54.1 and 18.7%, respectively on 2014, compared with 51.6 and 16.8% respectively on 2010 [4]. On the other hand, country-specific data published by the National Health and Nutrition Survey in 2012, indicate that the prevalence of overweight in children under 5 years has increased from 4.2% in 1986 to 8.6% in 2012. In the case of scholar children, a combined national prevalence of 29.9% between overweight and obesity was found, which means that 3 out of 10 students in the country present overweight and/or obesity problems [5].

Different strategies have been developed to reduce the morbidity and mortality that these diseases cause globally, among them, the implementation of policies that promote healthy nutritional practices, as well as the promotion of nutritional labeling of products as a measure of information and health promotion to the population [6].

Nutritional labels provide the energetical and chemical content of processed foods for the consumer. Moreover, these labels are used to raise awareness and promote appropriate nutritional habits in people. This tool could be useful if the consumers understand and use the information for the purchase and consumption of the products [7].

Regarding labels, those that emphazise on nutritional facts rather than schemes and colors are best undestood [8], however, nutritional information based on colors showed greater impact on consumers, helping them choosing their products on a better way [9]. Moreover, the more complex the label is, the most difficult to understand its content was [10, 11], althoug no significant differences were found between label schemes and their understanding level by the consumer [12].

Thus, the Nutritional Traffic Light Label (NTLL) is the most effective strategy in terms of guiding consumers towards healthier consumption, compared to other labeling systems [8, 13]. One of the first countries to implement this scheme was the United Kingdom, which by 2006 through the Food Standards Agency (FSA) implemented this strategy as a voluntary measure for the food industry, in order to help consumers to understand the nutritional information and thus make better decisions when making their purchases [14]. The greener the nutritional traffic light, the healthier its content [15], consequently, this initiative incresead the demand for products with healthier composition and influencing changes in eating behavior’s with real consequences in people’s diet [16].

In Ecuador, the Ministry of Public Health, on August 29, 2014 issued the “Health Regulation for Processed Food Labeling”, which includes the mandatory use of NTLL. The objective of the Ministry was to “regulate and control the labeling of processed foods, to guarantee the constitutional right of individuals to receive, clear, precise and non-misleading information about the content and characteristics of these foods, allowing consumers to take the right choice for their acquisition and consumption” [17].

However, there is no evidence about the effectiveness that this regulation has had as a public health strategy to alleviate and prevent the health problems mentioned above. The present study aims to evaluate the use and knowledge of nutritional traffic light label and its effectiveness as a public health promotion strategy in an urban population from Quito, Ecuador.

## Methods

In a cross-sectional study, conducted during the second semester of 2015, an urban population from Quito, Ecuador was evaluated about knowledge, understanding and comprehension of the NTLL. In addition, it sought to contrast the referred versus the observed use of the NTLL, to find out if they are related to the NTLL knowledge and more importantly, to compare and verify if the people’s referred use showed relationship with the real use of the NTLL.

The study population comprised a sample of 73 participants based on an estimated error of 5% and a probability of 95%. Participants who met the inclusion criteria proposed for the study (Table 1),were recruited in two supermarkets located at different socio-demographic and economic locations in the urban area of Quito, Ecuador: (a) Supermarket A, located in a downtown area and financial center of the city; and (b) Supermarket B, located in the peripheral area of the city.

**Table 1:** Participants’ exclusion criteria

The participant or someone at his household or direct family members, are currently under some nutritional regime (diet)	
The participant or someone at his household or direct family members suffers or has a history of any of the following: • Cardiovascular Disease (Hypertension, Heart Failure, Hypercholesterolemia, Myocardial Infarction, Atherosclerosis, etc.). • Metabolic Diseases (Diabetes Mellitus, Hypo / hyperthyroidism, Obesity, Overweight). • Eating disorders (anorexia, bulimia, etc.). • Kidney diseases or other chronic diseases (eg. cancer).	

Prior to any intervention, participants signed an informed consent form approved by the Bioethics Committee of the Universidad San Francisco de Quito, Ecuador. The potential participants were randomly chosen, at the supermarket and they had a shopping car filled with products at least by half. All participants were waiting on the payment line.

The first part of the study was based on a questionnaire focused on (1) demographic data; (2) knowledge, understanding and comprehension of the NTLL; (3) factors that influence the customer when making his/her purchase; and (4) subjective review of the pattern and trend of the participant’s purchase.

Self-knowledge was a dicotomic question (yes/no) but “level of knowledge” was assessed thourgh four questions and ranged as high (4/4), medium (3/4) or low (less than 3). Use of NTLL was evaluated with two dicotomic (yes/no) questions [(a) did you check NTLL during your purchase?, and (b) did the NTLL influence your purchase?], a four item Likert’s scale (always/sometimes/very few times/never) for the question did you based your purchases on the NTLL? and three dicotomic (use/do not use) questions about self use of NTLL. In Ecuador, the NTLL consists of a scheme that values three characteristics or nutrients of the products: fats, sugars and salt. Each one of them is represented by one of 3 colors defined as: high, medium and low content (red, yellow and green, respectively). In the current investigation, every product purchased by a participant was classified as “healthy” (no single red light on its label) or “non-healthy”.

The second part of the study was an observation of the participant’s purchasing pattern, which was done by taking individual photographs of the NTLL of each of the products, once they were registered by the cashier and prior to their package. Then, this data was analyzed as the objective part of the study to classify participants in levels depending on the amount of healthy (no red labels) products, as follows: “high” (> 74%); “medium” (50–74%); “low” (25–49%) and very low (< 25%) of their shopping list. Finally, global objective use of the NTLL was considered if at least 50% of the shopping list was “healthy”.

### Data analysis

The survey questions, as well as the information collected from the photographs were recorded and analyzed using the IBM SPSS Statistics, v20. Continuous variables (e.g.age) are presented as mean and standard deviation (SD). For categorical variables, Chi square or Fisher’s Exact test were used depending of the count. Finally, variables with a statistical significance at at α = 0.1 were included in multivariate logistic regression models adjusted for possible confounding factors (age, gender and educational level) to obtain OR and a *p*-value less than 0.05 was considered as significant.

## Results

The mean age of the participants was 38.4 SD 10.5 years old, the majority were female (58.6%, *n* = 41) and 90.4% (*n* = 66) had a high educational level (college or university).

Regarding subjective data, the majority (88.7%, *n* = 63) of participants indicated they knew about the NTLL. It is important to note that the majority (59.7%, *n* = 43) of participants mentioned they did not check the NTLL to do their purchase and for 78.1% (*n* = 57) the NTLL did not influence their purchase. In the same sense, 36.1% (*n* = 26) participants mentioned that their purchase is based on the NTLL “very few times”, 30.6% (*n* = 22) “sometimes” and only 9.7% (*n* = 7) indicated that they “always” based their purchase on it. Finally, 72.6% (*n* = 53) of the participants do not use the NTLL.

Also, the most determinant buying factor when grocery shopping were “brand” and “price” with 58.6 and 30% of participants respectively, while “nutrition/health” was only 7.1% of responses. Finally, for 80.6% of participants their purchase was “healthy” or “very healthy” for 12.5% of participants. Only 6.9% of participants recognized their purchase was “non-healthy”.

Regarding objective data, 2805 products were anotated with a median of 35 products per participant (range 11 to 143). Of those, healthy products (no red lights) were categorized in “low” and “medium” amount in 61.6 and 24.7% of the total, respectively. On the other hand, red lights (no-healthy) products were found at the “medium” and “low” levels with 60.3 and 20.5% of products, respectively.

88.7% of the participants indicated knowing about the NTLL. Most participants (52.1%) had a high level of knowledge of the NTLL, 32.9% had an average level of knowledge and only 15.1% had a low level of knowledge.

Analysis of the knowledge of the NTLL versus the subjective and objective variables, showed a significant association between knowledge of the NTLL and educational level. Thus, 91.8% of those who defined themselves as knowing the NTLL, showed to have a higher educational level.

Referred (subjective) use of the NTLL was almost equal to the observed use of it (27.4% vs. 28.4% of participants, respectively). Only “purchase factor” had a significant association (*p* = 0.02) with respect to the referred use. In fact, 87.8% of the participants who answered NO to the referred use of the NTLL mostly take into account brand of the product when making their purchases. Similarly, 57.1% of the participants who reported NOT using the NTLL also referred taking into account the price of the product at the time of their purchases.

There was no association between knowledge of the NTLL and the observed use of non-healthy products, the referred use and the supermarket, gender and educational level.

Multivariate logistic analysis showed a significant (*p* = 0.04) association between “does NTLL influence your purchase” and the observed use of the NTLL (OR 3.39 95% CI 1.06–10.79). In addition, an association (*p* = 0.04) was found between “NTLL knowledge” and observed use when it was adjusted by the supermarket (OR 4.26 CI 95% 1.08–16.76). No associations were found in the other crosses or with any of the other adjusted models.

## Discussion

In the present study, most of participants (88.7%) knew about the NTLL, however only 52.1% had a high level of knowledge of it. In this sense, nutritional labels are designed to provide simplified and concise information to customers, but it is still on the consumer hands, to correctly interpret and understand such information, being that an essential requirement for the final buying decision [16].

A previous study to evaluate the understanding of nutritional labels showed that approximately two thirds of the participants were able to identify the caloric content and daily values of the label [18], and the Canadian Council of Food and Nutrition survey reported that 80% participants felt confident about their understanding of nutritional labels [18]. Similarly, other studies suggested that participants based their decisions on the nutritional information provided by the product rather than their own knowledge about health and nutrition [19]. The use of the NTLL decreased the preference of hight fat and sugar content products [20], and the red color on the label was less probable to be catalogued as healthy [21].

Related to our results, the European Food Information Council reported that only 1 in 4 consumers looked for and used the NTLL while doing their shopping. In addition, observation of the NTLL by the consumer is in most cases by accidental exposure to the NTLL information in a product [22, 23].

The present study found that regarding the referred or subjective use of the NTLL, 59.7% participants did not look at it when making their purchase, which correlates to the 78.1% saying the NTLL did not influence their purchase. In addition, 72.6% of participants do not use the NTLL based on subjective data while 71.6% of participants do not use it based on the objective data. It was found also that for healthy products, most people bought medium and low amounts; however, this variable did not show significant relevance regarding knowledge of the NTLL.

Our study sought to discriminate differences in the use and understanding of the NTLL by making a socieconomic stratification, for which we conducted surveys in two different supermarkets. We also differentiated and analyzed for possible confounding factors such as gender, age and educational level. The present study did not find any association between socioeconomic status (supermarket), gender or age when compared with the knowledge and use of the NTLL. However, an association was found regarding the knowledge of NTLL and the educational level, which tends towards the highest level of education related to a positive knowledge of the NTLL.

In agreement with our results, a study in Spain showed based on subjective data that 41.4% of the participants knew about the NTLL, 61.5% of which stated that the NTLL seemed useful and 31.4% referring a usual use of it. In addition, the study also showed that 18.6% of the participants correctly understood the NTLL information [24].

Finally, in the case of Ecuador, there is only one previous study showing that the subjective use of the label was generally low, but higher among mestizos compared to indigenous people, owing mainly to differences in education and limited health and nutrition related knowledge in the indigenous population [25], similar to our study. Moreover, the latest official report on the strategy in the country, have concluded that in order to be successful, this strategy should also be supported by other public and private health promotion regulations, informative and educational campaigns directed both to the consumer and the producer, as well as the promotion of other healthy habits among the population [26].

Our study has several strenghs, among them, the contrast between reported and observed used of the NTLL, but we also recognize its main limitation is the lack of generalizability of the findings at a country-level.

## Conclusion

In this urban population, the level of knowledge of the NTLL was relatively high, however, the observed use of the NTLL was low and it was associated with the socioeconomic and educational status of the participants. This study puts in evidence that a successful strategy implementation does not only depends on good regulation, but also requires customer acceptance and involvement. It would be interesting to replicate this model in any other country with NTLL in place.
